# An In-Depth Exploration of the Autoantibody Immune Profile in ME/CFS Using Novel Antigen Profiling Techniques

**DOI:** 10.3390/ijms26062799

**Published:** 2025-03-20

**Authors:** Arnaud Germain, Jillian R. Jaycox, Christopher J. Emig, Aaron M. Ring, Maureen R. Hanson

**Affiliations:** 1 Department of Molecular Biology and Genetics, Biotechnology Building, Cornell University, Ithaca, NY 14853, USA; ag297@cornell.edu; 2 Division of Translational Science and Therapeutics, Fred Hutchinson Cancer Center, Seattle, WA 98109, USA; jillian.jaycox@yale.edu (J.R.J.); aaronring@fredhutch.org (A.M.R.); 3 Department of Immunobiology, Yale School of Medicine, New Haven, CT 06510, USA; 4 Augmenta Bioworks, Inc., Menlo Park, CA 94025, USA; chris@augbio.com

**Keywords:** ME/CFS, immunity, autoantibodies, antiviral antibodies, herpesvirus, GPCR, β-adrenergic receptors, muscarinic receptors

## Abstract

Myalgic encephalomyelitis/chronic fatigue syndrome (ME/CFS) is a debilitating disorder characterized by serious physical and cognitive impairments. Recent research underscores the role of immune dysfunction, including the role of autoantibodies, in ME/CFS pathophysiology. Expanding on previous studies, we analyzed 7542 antibody–antigen interactions in ME/CFS patients using two advanced platforms: a 1134 autoantibody Luminex panel from Oncimmune and Augmenta Bioworks, along with Rapid Extracellular Antigen Profiling (REAP), a validated high-throughput method that measures autoantibody reactivity against 6183 extracellular human proteins and 225 human viral pathogen proteins. Unlike earlier reports, our analysis of 172 participants revealed no significant differences in autoantibody reactivities between ME/CFS patients and controls, including against GPCRs such as β-adrenergic receptors. However, subtle trends in autoantibody ratios between male and female ME/CFS subgroups, along with patterns of herpesvirus reactivation, suggest the need for broader and more detailed exploration.

## 1. Introduction

Myalgic encephalomyelitis/chronic fatigue syndrome (ME/CFS) is a complex and debilitating disorder characterized by persistent and profound fatigue, headaches, sensitivity to light and sound, cognitive impairment, muscle pain, and post-exertional malaise [1]. Despite the profound impact on the lives of millions worldwide, the precise cause of ME/CFS remains elusive, making both diagnosis and treatment particularly challenging. However, recent research highlights the potential role of immune dysfunction in the disease [2,3,4,5,6,7,8,9]. A major hypothesis is the involvement of autoantibodies (AAb), which target the body’s own tissues or organs and have been implicated in numerous autoimmune diseases like lupus, multiple sclerosis, and rheumatoid arthritis—conditions that share some symptoms with ME/CFS [10]. Some individuals may produce pathogenic AAb that lead to chronic inflammation and tissue damage, potentially contributing to the development or exacerbation of ME/CFS symptoms.

The involvement of AAb in ME/CFS is supported by two distinct clinical interventions leading to significant symptom improvement in a subset of ME/CFS patients. One implicates B-cell (AAb producers) depletion therapy using rituximab, where approximately 60% of patients self-reported moderate to major improvements in fatigue scores followed by a relapse in symptoms upon B-cell regeneration [11,12]. However, larger follow-up studies on the long-term benefits of rituximab treatment failed to show any benefit compared to placebo [13,14]. The second intervention involved immunoadsorption therapy, filtering out immunoglobulin G (IgG) AAb using an IgG-binding column, yielding temporary symptom relief similar to that observed in rituximab trials, although neither of these studies was placebo-controlled [15,16].

Based on ME/CFS symptoms related to central nervous system function, an early study investigated several AAb against neurotransmitter G protein-coupled receptors (GPCRs) and found a significant increase in AAb targeting M1-muscarinic receptors [17]. Subsequent studies identified elevated levels of AAb in ME/CFS patients against β1/β2/β3-adrenergic and M3/M4-muscarinic receptors, which are crucial for the regulation of autonomic and cardiovascular functions [18,19]. Such dysregulation could explain orthostatic intolerance, another common feature of ME/CFS.

While much research has concentrated on AAb targeting the GPCRs, the AAb immune profile is much more extensive. A recent study investigated 33 naturally occurring AAb against neural and non-tissue-specific autoantigens (with several GPCRs overlapping with prior studies) in 11 ME/CFS patients [20]. The study linked a disturbed AAb network signature with symptom severity in ME/CFS while also noting the presence of a similar AAb network signature in their 11 healthy controls. Notably, naturally occurring AAb against GPCRs are present not only in healthy individuals but also in conditions such as systemic sclerosis, Alzheimer’s disease, ovarian cancer, and COVID-19 [21,22]. Additionally, natural antibodies, which are autoantibodies produced constitutively in the absence of antigens, have been shown to play important roles in homeostasis and the regulation of disease processes, for example, through the clearance of cellular debris and oxidatively damaged proteins [23,24], suggesting their significance in immune function and other physiological processes like insulin regulation, with variations depending on age and sex [25,26]. Finally, while autoantibodies are traditionally thought of as drivers or markers for diseases, many examples of autoantibodies acting as beneficial regulators of patient physiology also exist; for example, patients with the autoimmune disease lupus that produce neutralizing AAb against interferon have milder disease than those without [27].

Our study dramatically expands the scope of AAb analysis by examining a dataset consisting of 7542 antibody–antigen interactions in ME/CFS patients, using two innovative techniques. One method, developed by Oncimmune Ltd. and Augmenta Bioworks Inc., analyzed a panel of 1134 antibodies using Luminex, a high-throughput bead-based platform, enabling the simultaneous analysis of multiple AAb in a single sample using flow cytometry. The second method, Rapid Extracellular Antigen Profiling (REAP), is a yeast display library platform that allows for the assessment of antibody reactivity against 6183 human extracellular proteins, peptide epitopes, and 225 viral antigens. The cohorts included 103 and 164 subjects, respectively, with 95 individuals in common, covering both ME/CFS patients and age- and sex-matched healthy controls.

Despite the extensive scope of the data, no significant differences in AAb levels between ME/CFS patients and healthy controls were observed, including none in AAb to previously studied β-adrenergic receptors. However, when females and males were separated in the Oncimmune/Augmenta dataset, subtle differences emerged when analyzing AAb ratios between patients and controls. Additionally, the viral REAP data revealed elevated herpesvirus antibody levels, echoing findings of herpes viral reactivation in other studies in ME/CFS and in long COVID [28,29,30].

## 2. Results

### 2.1. Study Design and Cohort Characteristics

Our study included a total of 172 unique participants, divided into two cohorts with approximately 92% overlap. For the Oncimmune/Augmenta dataset, we utilized 103 plasma samples, while the REAP dataset was generated using 164 serum samples. The demographic information for both cohorts is summarized in Table 1, indicating reasonable age- and BMI-matching while demonstrating the significantly lower functional status of ME/CFS subjects, with lower scores for the Bell disability scale. The ME/CFS subjects fulfilled the Canadian consensus criteria for ME/CFS diagnosis [31]. Subject selection was performed by physicians, and blood collection was carried out at Ithaca College (Ithaca, NY, USA), Weill Cornell Medicine (NYC, NY, USA), and Workwell Foundation (Ripon, CA, USA). All ME/CFS subjects developed ME/CFS before 2020.

### 2.2. AAb Profiling Reveals No Significant Difference Between ME/CFS and Controls

Oncimmune has developed panels of human autoantigens for use in cancer and autoimmune diagnostics [32,33,34,35,36,37,38]. Augmenta Bioworks supplemented the Oncimmune 1126 antigen panel with an additional 11 antigens associated with viral infection and autoimmune disease. The latter 11 antigens were measured simultaneously with 122 human pathogens discussed previously [39]. In total, we measured AAb in plasma samples from 103 participants—including 59 ME/CFS and 44 controls with 80% and 66% females, respectively—resulting in a dataset of 1134 quantified AAb (Supplementary File S1). To investigate differences in AAb levels between ME/CFS patients and controls, we applied a linear model (AAb ~ phenotype * sex + age + BMI + site) to adjust for confounding factors. This analysis was performed on the entire cohort and separately for female and male subgroups (in which sex was excluded from the model) to explore potential sex-specific disparities in AAb profiles. Multiple comparisons were accounted for with the Benjamini–Hochberg (BH) adjustment.

**Figure 1:**
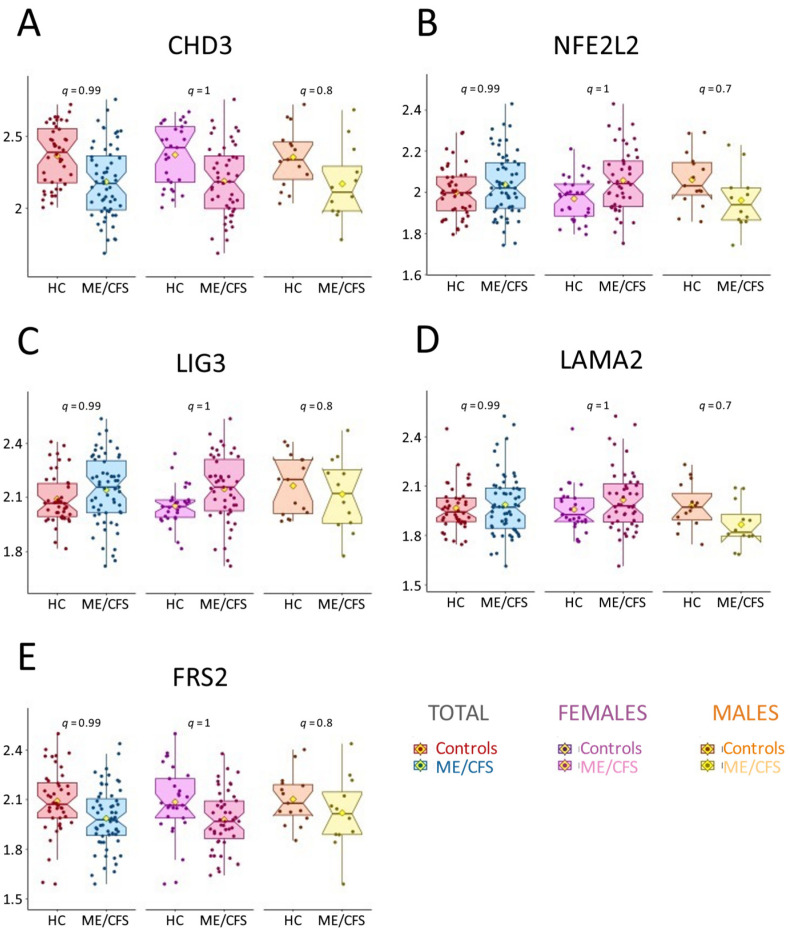
Box plots of (**A**) CDH3, (**B**) NFE2L2, (**C**) LIG3, (**D**) LAMA2, and (**E**) FRS2 AAb measured for the total cohort and for the female and male subgroups; HC = healthy controls. The y-axis values are the log2-transformed values. The yellow diamonds represent the means. The *p*-values and *q*-values from the linear model are shown for each subgroup. Each dot represents one individual.

We found no significant differences between ME/CFS patients and controls in any of the AAb assessed by our assay, with no q-value lower than 0.68 for any of the subgroups (using the BH adjustment) (Supplementary File S2). To illustrate the data distribution of our dataset, the top five examples starting from the lowest uncorrected *p*-values for the total cohort are shown in Figure 1. They include CHD3 (Chromodomain-helicase-DNA-binding Protein 3), a component of the histone deacetylase NuRD complex (Figure 1A); NFE2L2 (Nuclear factor erythroid 2-related factor 2), a transcription factor involved in oxidative stress response (Figure 1B); LIG3 (DNA ligase 3), part of a DNA-repair complex functioning in the nucleus and mitochondria (Figure 1C); LAMA2 (Laminin subunit alpha-2), involved in the attachment, migration, and organization of cells into tissues (Figure 1D); and FRS2 (Fibroblast growth factor receptor substrate 2), which plays an important role in signaling pathways that include PIK3R1 (Figure 1E).

### 2.3. Sex-Specific Trends in AAb Abundance: Lower Levels in Male ME/CFS Patients

**Figure 2:**
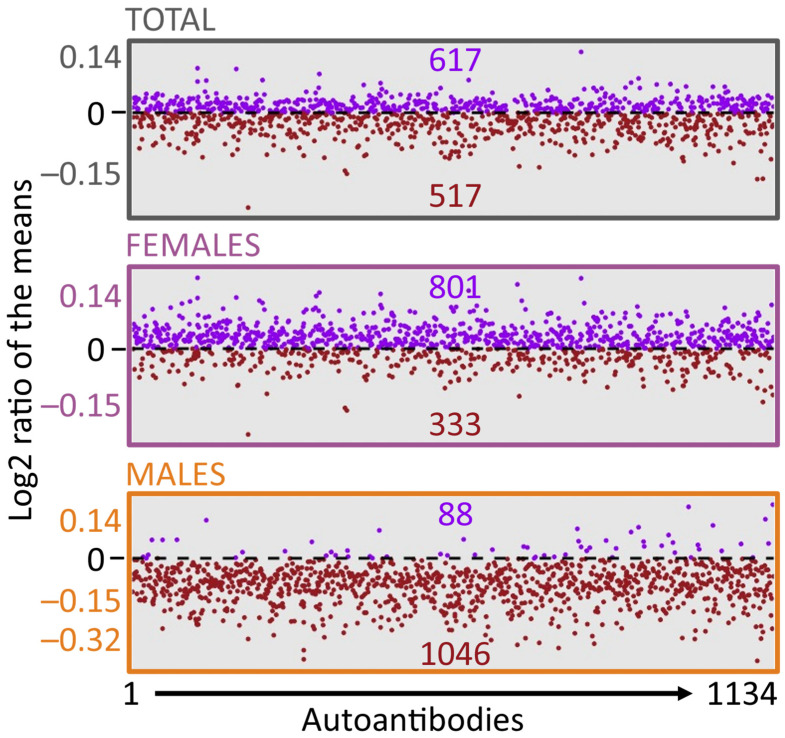
Dot plot of the log2 ratio of the means of AAb for the total cohort and the female and male subgroups. The *x*-axis represents the 1134 AAb measured through Luminex assays by Oncimmune and Augmenta Bioworks, ordered alphabetically from 1 to 1134. The *y*-axis is the log2 ratio of the means, with ‘0’ meaning no fold change, ‘<0’ indicating lower levels of AAb (brown), and ‘>0’ indicating higher levels of AAb (purple) in ME/CFS compared to controls.

A notable feature of the dataset is the tendency for a lower abundance of AAb in the male ME/CFS subgroup compared to the controls, as briefly illustrated in some of the panels of Figure 1. This pattern was observed across much of the dataset, prompting us to calculate the ratio of mean AAb levels for each antibody in the three subgroups: total, females, and males. Figure 2 presents the results for all 1134 AAb, revealing that 92% of AAb show lower mean levels in the male ME/CFS cohort, whereas 71% exhibit higher levels in the female ME/CFS cohort compared to the controls. It is important to note, however, that the log2 value of many of these ratios of the means are close to ‘0’, indicative of minimal changes in AAb levels.

### 2.4. ME/CFS Patients Lack Public Autoantigens Against the Human Exoproteome

AAb reactivity against extracellular human proteins was assessed using Rapid Extracellular Antigen Profiling (REAP), a validated high-throughput method that allows for the measurement of antibody reactivity against 6183 human extracellular and secreted proteins (Supplementary File S3).

**Figure 3:**
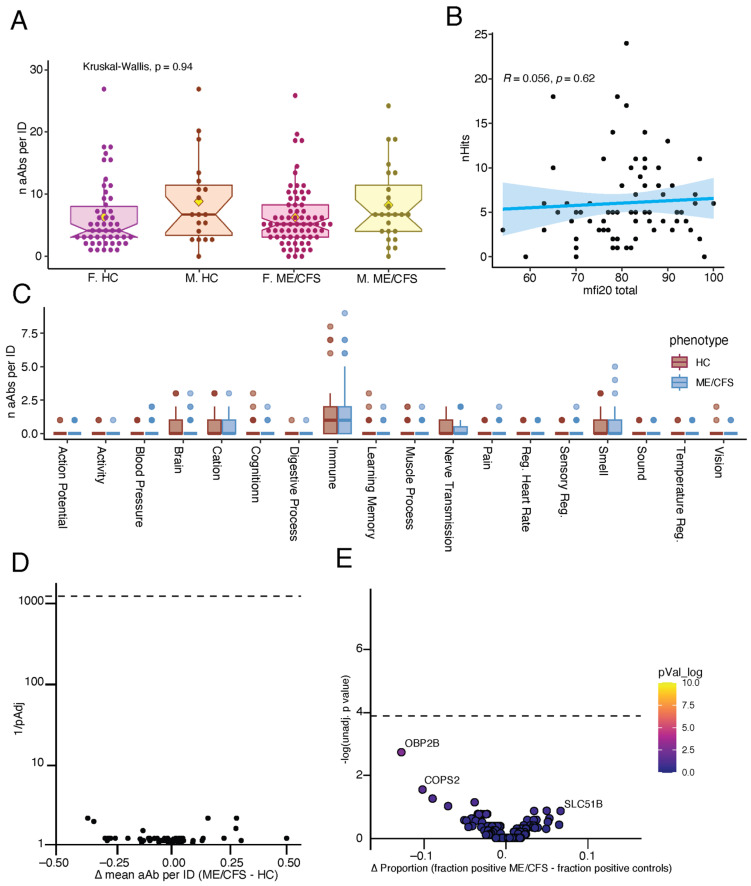
ME/CFS patients lack public autoantigens against the human exoproteome. (**A**) The number of AAb reactivities per individual (ID) by group. REAP reactivity is defined as a REAP score ≥ 1. Significance was assessed using Kruskal–Wallis tests. For the box plots, the central lines indicate the group median values, the top and bottom lines indicate the 75th and 25th percentiles, respectively, and the whiskers represent 1.5× the interquartile range. The yellow diamonds represent the means. Each dot represents one individual. F.HC: Female, healthy control; F.ME/CFS: Female, ME/CFS; M.HC: Male, healthy control; and M.ME/CFS: Male, ME/CFS. (**B**) The relationship between the number of AAb reactivities per individual and MFI-20. Correlation was assessed using Spearman’s correlation. The blue line shows the linear regression, and the shading shows the 95% CIs. Each dot represents one individual. (**C**) Grouped box plot depicting the number of AAb reactivities per individual in the listed GO Process domain. Statistical significance was assessed by unpaired Wilcoxon rank-sum tests and adjusted for multiple comparisons using FDR (Benjamini–Hochberg) correction. All comparisons are non-significant. Colored box plots depict the 25th to 75th percentiles of the data, with the middle line representing the median, the whiskers representing 1.5× the interquartile range, and the dots depicting the outliers. (**D**) Assessment of differential AAb reactivity against specific tissues in participants with ME/CFS versus controls. Significance was assessed by unpaired Wilcoxon rank-sum tests. The y-axis shows −log_10_-transformed unadjusted *p*-values; the Bonferroni-adjusted significance threshold is indicated by a black dashed line. The *x*-axis shows the difference in the mean number of AAb against a given tissue in ME/CFS minus controls. Each dot represents one tissue. (**E**) Assessment of the frequency of individual AAb reactivities in participants with ME/CFS and control individuals. Significance was assessed using Fisher’s exact tests. The *y*-axis shows −log_10_-transformed unadjusted *p*-values; the Bonferroni-adjusted significance threshold is indicated by a black dashed line. The *x*-axis shows the difference in the proportion of AAb-positive individuals in each group. Each dot represents one AAb reactivity.

AAb reactivity profiles among participants with ME/CFS demonstrated a variety of private reactivities against diverse human autoantigens (Figure S1). However, there was no difference in the total number of AAb reactivities, as defined by a REAP score greater than or equal to 1, per individual between cases and controls (Figure 3A), and there was also no correlation between the number of reactivities and overall disease severity, as measured by the multidimensional fatigue inventory—MFI-20 (Figure 3B).

Given previous findings of elevated functional GPCR AAb in ME/CFS, AAb reactivities were aggregated into clusters using a manually curated GO Process list relevant to symptoms experienced by patients with ME/CFS. There were no significant differences in the number of reactivities per individual between ME/CFS and controls across any of the categories (Figure 3C).

Next, we used Human Protein Atlas mRNA expression data to categorize REAP antigens into 44 different tissue categories by mRNA expression. We performed an unbiased analysis to assess the differential targeting of specific tissues by AAb in ME/CFS patients versus controls; we found no significant enrichment in AAb against any tissue (Figure 3D).

Finally, we assessed the enrichment of individual AAb reactivities in ME/CFS patients versus controls. We found no individual AAb reactivities that were significantly more common in ME/CFS patients compared to controls (Figure 3E).

### 2.5. ME/CFS Patients Display Elevated Humoral Responses to Herpesviruses

Next, we used REAP to assess humoral reactivity against 225 surface proteins of human viral pathogens. We detected reactivity against 54 different viral proteins (Figure S2A) amongst our cohort of 95 ME/CFS patients and 69 controls.

**Figure 4:**
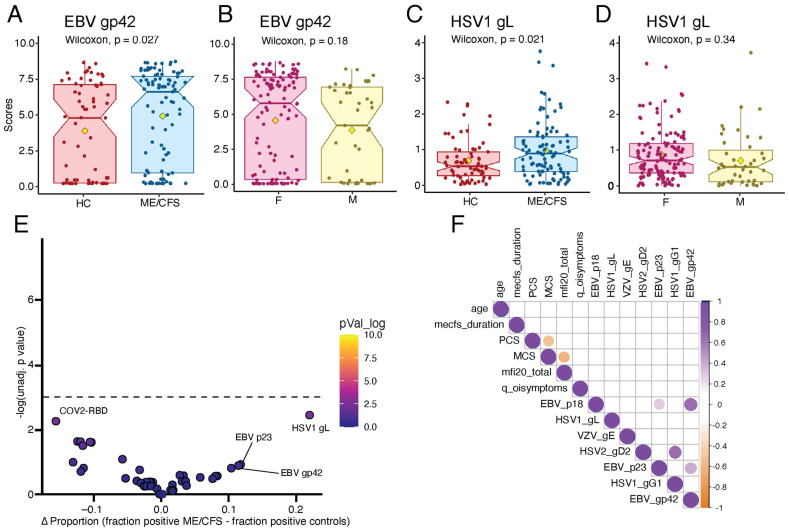
ME/CFS patients display elevated humoral responses to herpesviruses. (**A**,**B**) EBV gp42 REAP scores of healthy controls versus ME/CFS patients and female (F) versus male (M) ME/CFS patients, respectively. Each dot represents one individual. Statistical significance was assessed by unpaired Wilcoxon rank-sum tests. The central lines indicate the group median values, the top and bottom lines indicate the 75th and 25th percentiles, respectively, and the whiskers represent 1.5× the interquartile range. The yellow diamonds represent the means. (**C**,**D**) HSV1 gL REAP scores of healthy controls versus ME/CFS patients and female (F) versus male (M) ME/CFS patients. Each dot represents one individual. Statistical significance was assessed by unpaired Wilcoxon rank-sum tests. The central lines indicate the group median values, the top and bottom lines indicate the 75th and 25th percentiles, respectively, and the whiskers represent 1.5× the interquartile range. The yellow diamonds represent the means. (**E**) Assessment of the frequency of binary viral reactivity (REAP score > 1) in participants with ME/CFS and healthy control individuals. Significance was assessed using Fisher’s exact tests. The *y*-axis shows −log_10_-transformed unadjusted *p*-values; the Bonferroni-adjusted significance threshold is indicated by a black dashed line. The *x*-axis shows the difference in the proportion of antibody-positive individuals in each group. Each dot represents one antibody reactivity. (**F**) Correlation matrix depicting pair-wise correlations between 13 labelled variables. Correlation was assessed using Spearman’s correlation tests. Colored cells indicate statistically significant results after *p*-value correction using the Bonferroni method. Cell color depicts the correlation coefficient (R).

Interestingly, ME/CFS patients displayed higher REAP reactivity against EBV gp42, a component of the EBV fusion receptor (Figure 4A), with no significant difference in reactivity observed between ME/CFS males and females (Figure 4B). ME/CFS patients showed a non-significant trend for elevated REAP reactivity toward other EBV-associated antigens, including capsid antigens p23 (Figure S2B) and p18 (Figure S2C). We also observed increased REAP reactivity against HSV1 glycoprotein gL (Figure 4C) in ME/CFS patients. Again, there was no difference in REAP score for this antigen observed in male versus female patients (Figure 4D). We found no difference in REAP reactivity in patients versus controls against other HSV1- and HSV2-associated antigens included in the REAP library (Figure S2D,E).

Given the differences in REAP score magnitude for several herpesvirus antigens, we then performed an unbiased binary enrichment to assess differences in “seropositivity” between cases and controls (Figure 4E). Reactivity toward HSV1 gL was most differentially enriched in ME/CFS patients, with approximately 41% of ME/CFS patients and only 19% of controls displaying REAP reactivity to this antigen; however, this result was not statistically significant.

Given the hypothesized role in herpesvirus reactivation in driving ME/CFS pathology, we assessed the correlation between herpesvirus antigen reactivity by REAP and demographic metrics and symptom severity, as measured by the 36-item short-form survey instrument (SF-36) mental component summary score (MCS), physical component summary score (PCS), and MFI-20. We did not observe any significant correlations between herpesvirus antigen REAP scores and symptom severity or demographic metrics (Figure 4F). For example, there was no correlation between EBV gp42 REAP score and either ME/CFS disease duration (Figure S2F) or MFI 20 (Figure S2G). Interestingly, we observed correlations between REAP scores for herpesvirus antigens for a given virus, suggesting that a subset of patients may have elevated reactivity against all EBV or all HSV1 antigens; for example, REAP scores against EBV gp42 and EBV p18 were highly correlated (R = 0.63, *p* = 9.8 × 10^−12^; Figure S2H).

### 2.6. AAb Results Do Not Align with Previous Findings

Previous research has implicated that AAb that target GPCRs are involved in central and autonomic nervous system function, for example, adrenergic, muscarinic, serotonin, dopamine, olfactory, and opioid receptors, in ME/CFS pathogenesis [17]. While some studies have suggested that certain AAb levels may be elevated in ME/CFS patients, inconsistencies exist regarding the significance of these differences, and findings vary regarding how AAb levels compare between healthy subjects and ME/CFS patients.

**Figure 5:**
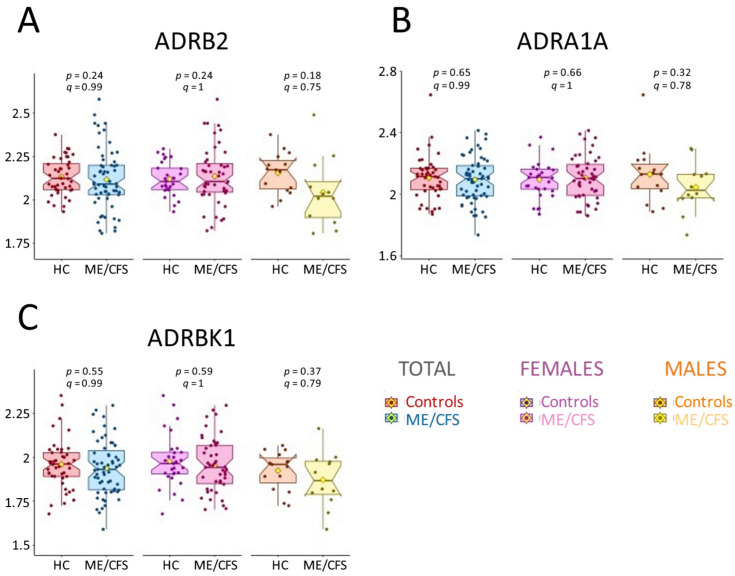
Box plots of AAb measured for the total cohort and for female and male subgroups for (**A**) ADRB2, (**B**) ADRA1A, and (**C**) ADRBK1; HC = healthy controls. The *y*-axis values are the log2-transformed values provided by Oncimmune/Augmenta. The yellow diamonds represent the means. The *p*-values and q-values from the linear model are shown for each subgroup. Each dot represents one individual.

The Oncimmune/Augmenta technique included ADRB2 (β2-adrenergic receptor), ADRA1A (α1A-adrenergic receptor), two adrenergic members of the GPCR family, and ADRBK1 (also known as GRK2: G-protein-coupled receptor kinase 2), which is essential in regulating ADRB2 and ADRA1A function [40]. Figure 5 presents the box plots for these three AAb for the total cohort and the female and male subgroups. The corresponding *p*-values and q-values indicate no significant difference between patients and controls, contrasting with previous studies that reported elevated levels of ADRB2 in ME/CFS [18,19]. Interestingly, within the male cohort, the trend is opposite, with lower AAb levels in the ME/CFS patients compared to controls, as shown in Figure 2.

In addition to the AAb identified in the Augmenta Bioworks dataset (Figure 5), several other AAb previously studied in ME/CFS subjects were evaluated using REAP.

These included two extracellular domains (ECDs) of OPRM1 (opioid receptor mu 1), HTR1A (5-hydroxytryptamine receptor 1A, a serotonin receptor) and two ECDs of DRD2 (dopamine receptor D2) [17]. No ME/CFS patients displayed detectable reactivity to these proteins. The REAP library also included a broad range of adrenergic and muscarinic receptor AAb (ADRA1A, ADRA1B, ADRA2A, ADRA2B, ADRA2C, ADRB1, ADRB2, ADRB3, CHRM1, CHRM2, CHRM3, and CHRM4). Despite being part of our analysis, we did not detect AAb reactivity against these proteins, apart from one female ME/CFS subject with REAP reactivity against the fourth ECD of ADRA2B.

## 3. Discussion

Our study offers unprecedented profiling of AAb against 7542 antigens in ME/CFS patients and controls using two complementary advanced approaches, namely, Luminex measurements and REAP. Specifically, the Oncimmune/Augmenta dataset generally focuses on the discovery of AAb against intracellular proteins, while REAP includes extracellular proteins and domains of extracellular viral proteins. The complementarity lies in the exoproteome, typically reflecting proteins that are more accessible to immune surveillance, whereas intracellular proteins may only become targets of the immune system in contexts like cell damage or death.

The major outcome of our analysis is the lack of significant differences in AAb levels between ME/CFS patients and controls for all individual AAb assessed (Figure 1 and Supplementary Files S1 and S3). However, we observed a general trend with higher AAb levels in the female cohort and an inverse trend in the male cohort, as depicted in Figure 2. Such a sex-based trend has been reported by our group, using the same cohort as the Oncimmune/Augmenta cohort used here, for an anti-pathogen antibody panel provided by Augmenta Bioworks [39]. These sex-specific differences in humoral responses and autoimmunity have been well-documented, with women producing more robust humoral responses in general, possessing higher levels of IgG and a higher frequency of rheumatologic autoantibodies, and being disproportionately affected by autoimmune disorders [41,42,43,44,45,46]. Understanding these differences may offer valuable insights for developing sex-specific personalized treatments, potentially improving therapeutic outcomes.

A major challenge is the lack of reproducibility of findings on AAb levels in ME/CFS research, particularly those related to GPCR AAb that have been suggested to play a role in ME/CFS. For example, while some studies have reported significantly elevated levels of β2-adrenergic receptor (ADRB2) AAb in certain cohorts [18,19], others—including a different cohort within the same study—found no significant differences [19,47]. A recent comparison of patients with a post-COVID ME/CFS diagnosis versus patients with solely a long COVID diagnosis showed that both groups had lower ADRB2 levels than healthy controls [48]. One possible etiology for the discrepancy is that GPCRs are structurally complex proteins with seven transmembrane domains, four extracellular domains, and four intracellular domains; thus, producing these proteins recombinantly outside of their native cell membrane and assessing autoantibody binding in a standard assay, such as ELISA, is challenging and unlikely to have high fidelity. Indeed, the REAP library displays GPCR extracellular domains (ECDs) separately, and therefore, we are not able to detect certain GPCR autoantibodies; for example, we were not able to detect those that require contact with multiple epitopes on separate ECDs for binding. Along with discrepancies in protein production, differences in technology or protocols utilized to assess binding could generate disparate results. Finally, patient heterogeneity in ME/CFS is broad and may lead to conflicting results; for example, AAb prevalence may vary strongly with disease duration as immune responses vary with the stage of the disease. Factors such as age and sex are also likely to be contributors; older patients or those with longer disease duration may exhibit different immune profiles than younger patients or those in earlier stages.

Nevertheless, although we were not able to replicate previously published findings showing elevated GPCR AAb in ME/CFS patients, it is crucial to recognize the recent successful outcomes of immunoadsorption therapy (IA) interventions [15,16]. IA is designed to remove pathogenic AAb from the bloodstream by returning IgG-depleted plasma to subjects after adsorption on a column. While the reprieve in symptoms experienced by responders is a testament to the possible involvement of AAb in symptom severity, such interventions were targeted towards a class of antibodies and not restricted to the potential GPCR AAb. Given our results and the existence of discrepant reports of elevated and lower levels of ADRB2 AAb levels, as addressed in the previous paragraph, the success of IA in responding ME/CFS subjects could be attributed to other AAb that are not yet identified or through the clearance of IgG with inflammatory glycan patterns, for example, afucosylated IgG.

Finally, using REAP, we found elevated reactivity against specific EBV and HSV1 surface antigens (Figure 4). This is interesting given a large body of evidence associating herpesvirus reactivation with ME/CFS [28]. In both pre-2020 ME/CFS and long COVID, latent viral reactivation—particularly from viruses like Epstein-Barr virus (EBV) or Human Herpesvirus 6 (HHV-6)—has been hypothesized to trigger or exacerbate symptoms [48,49,50]. Interestingly, we previously reported similar viral reactivity trends in long COVID patients, although these differences were more pronounced and covered additional herpesvirus antigens [51]. Orthogonal validation of herpesvirus reactivation using ELISA showed a strong and significant correlation to REAP scores in these long COVID samples. One possibility for the differences in the magnitude of the findings between the two conditions is the relatively acute nature of the long COVID cohort (disease duration of approximate 1 year) versus our ME/CFS cohort, which had an average disease duration of 11.3 and 13.7 years for female and male patients, respectively. Overall, the parallels between herpesvirus reactivation in ME/CFS and similar findings in long COVID suggest that shared immune dysregulation mechanisms may be at play. However, the exact contribution of herpesvirus reactivation to disease pathophysiology remains uncertain, and our data showed significant differences in reactivity to proteins of only two of the eight strains measured, namely, HSV1 gL and EBV gp42, with much overlap between controls and patients (Figure 4). This raises the possibility that viral reactivation, while being an important factor in ME/CFS, may not uniformly impact all patients or may only play a role in the early stage of the disease, which was not represented by our cohort.

In conclusion, our study highlights the complexities of AAb research in ME/CFS, emphasizing the need for standardized methodologies and consideration of patient heterogeneity. Understanding the underlying factors contributing to these discrepancies, such as methodological differences, viral reactivation, and patient demographics, will be critical for advancing our knowledge of ME/CFS and its immunological underpinnings.

## 4. Materials and Methods

### 4.1. Blood Collection

Blood samples were collected from a vein in the antecubital fossa into EDTA tubes and conveyed to processing laboratories the same day. Plasma was isolated following centrifugation of blood at 500× *g* for 5 min at room temperature. For serum, blood was left to clot for at least 30 min and then spun at 1500× *g* for 5 min at room temperature. Both plasma and serum samples were immediately stored at −80 °C.

### 4.2. Serological Testing by Augmenta Bioworks 

This test was performed following methods previously described [39]. In short, a panel of Luminex xMAP beads was constructed by coupling beads to recombinant proteins, inactivated viruses, and cell cultures, using rigorous inactivation and coupling methods to ensure safety and specificity. Quality control measures, including protein quantitation and antibody detection assays, validated bead–antigen conjugations, while log2-transformed fluorescence intensity data were normalized and analyzed to assess antigen-specific antibody binding.

### 4.3. Oncimmune SeroTag Profiling Overview

The SeroTag^®^ multiplex workflow uses Luminex color-coded beads, which permits the multiplex measurements of the AAb reactivity towards 384 candidate antigens in one sample. Magnetic beads were employed to enable automated pipetting and washing steps. The antigen–bead mix was incubated with a sample, which may have contained AAb against the target protein. Bound AAb were detected using an anti-IgG-specific detection antibody, which was conjugated to the fluorescent reporter dye phycoerythrin (PE). A Luminex FlexMAP3D analyzer was used to identify and quantify each antigen–antibody reaction based on bead color and fluorescence intensity. The median bead fluorescence intensity (MFI) was then calculated and used for data analysis.

Serum samples were diluted to 1:100 in an assay buffer (PBS, 0.5% BSA, and 50% Low-Cross buffer—Candor Biosciences, Wangen, Germany). Diluted serum and beads were mixed and incubated at 4 °C to 8 °C in the dark. The bound AAb were detected by the addition of an anti-IgG-specific detection antibody, which was conjugated to the reporter dye phycoerythrin (PE). A Luminex FlexMAP3D analyzer was used to identify and quantify each antigen–antibody reaction based on bead color and fluorescence intensity.

### 4.4. Oncimmune/Augmenta Data Analysis

Eight of the missing values were replaced by the minimum value for that AAb. Univariate statistical analysis for each AAb was performed using a linear mixed model with disease status, age, BMI, site, and sex as fixed effects and subject as a random effect. The Benjamini–Hochberg (BH) method was used to correct for false discovery rate [52].

### 4.5. REAP Library Expansion

Briefly, the initial yeast library (Exo201), containing only extracellular domains > 49 amino acids in length, was generated as previously described [53,54]. REAP library expansion was performed as previously described [51].

Briefly, all extracellular domains of multi-pass membrane proteins greater than 15 amino acids and 225 extracellular viral antigens were synthesized by TWIST Bioscience, containing a 5′ sequence (CTGTTATTGCTAGCGTTTTAGCA) and 3′ sequence (GCGGCCGCTTCTGGTGGC) for PCR amplification. The oligo pool was PCR-amplified and transformed into yeast with barcode fragments, followed by barcode–antigen pairing identification. This new yeast library was then pooled with the initial library (Exo201) to generate the new version of the library (Exo205), which contained 6452 unique antigens.

### 4.6. REAP Protocol

Participant IgG isolation and REAP selections were performed as previously described [53,54]. Briefly, IgG was purified using protein G magnetic beads followed by adsorption to yeast to remove yeast-reactive IgG. The Exo205 yeast library was induced in a SGO-Ura medium, and yeast cells were washed and mixed with 10 μg of purified participant IgG for 1 h. Yeast cells were washed and incubated with 1:100 biotin anti-human IgG Fc antibody (BioLegend, San Diego, CA, USA, QA19A42) for 30 min. Yeast cells were washed and incubated with a 1:20 dilution of Streptavidin MicroBeads (Miltenyi Biotec, Bergisch Gladbach, Germany, 130-048-101) for 30 min. IgG-bound yeast were isolated by positive magnetic selection using the MultiMACS M96 Separator (Miltenyi Biotec, Bergisch Gladbach, Germany) according to the manufacturer’s instructions. Selected yeasts were resuspended in 1 mL SDO−Ura and incubated at 30 °C for 24 h; then, plasmid DNA was collected for NGS analysis using Zymoprep-96 Yeast Plasmid Miniprep kits or Zymoprep Yeast Plasmid Miniprep II kits (Zymo Research, Irvine, CA, USA, D2007). PCR was used to amplify a DNA sequence containing the protein display barcode on the yeast plasmid and to add Nextera i5 and i7 dual-index library barcodes (Illumina, San Diego, CA, USA). PCR products were pooled and run on a 1% agarose gel, and DNA corresponding to the band at 257 bp was cut. DNA (NGS library) was extracted using the QIAquick Gel Extraction Kit (Qiagen, Hilden, Germany, 28704). The NGS library was sequenced using the Illumina NextSeq 2000 system and a NextSeq 2000 P3 100 cycles sequencing kit (Illumina, 20040559) with 75 bp single-end sequencing.

### 4.7. REAP Data Analysis

REAP scores were calculated as previously described [53,54]. In brief, barcode counts were extracted from raw NGS data using custom codes, and counts from technical replicates were summed. Next, aggregate and clonal enrichment was calculated using edgeR70 and custom computer scripts. Aggregate enrichment is the log2-transformed fold change of all barcodes associated with a particular protein summed in the post-library relative to the pre-library, with zeroes in the place of negative fold changes. Log2-transformed fold change values for clonal enrichment were calculated in an identical manner, but barcode counts across all unique barcodes associated with a given protein were not summed. Clonal enrichment for a given reactivity was defined as the fraction of clones out of total clones that were enriched (log2 fold change ≥ 2). Aggregate (Ea) and clonal enrichment (Ec) for a given protein, a scaling factor (βu) based on the number of unique yeast clones (yeasts that have a unique DNA barcode) displaying a given protein, and a scaling factor (βf) based on the overall frequency of yeast in the library displaying a given protein were used as inputs to calculate the REAP score, which is defined as follows:

$$REAP\;score=Εa\times {\left(Εc\right)}^{2}\times \beta u\times \beta f$$

βu and βf are logarithmic scaling factors that progressively penalize the REAP score of proteins with low numbers of unique barcodes or low frequencies in the library and are described in detail previously [16].

Antigens with an average REAP score greater than 0.5 across all samples were defined as non-specific and were excluded from further analysis. AAb reactivities were defined as antigens with a REAP score greater than or equal to 1.

## Figures and Tables

**Table 1:** Study demographics.

	Oncimmune/Augmenta			REAP		
	ME/CFS	Controls	*p*-Value	ME/CFS	Controls	*p*-Value
Cohort (n)	59	44	NA	95	69	NA
Females/Males (n)	47/12	29/15	NA	71/24	50/19	NA
Age (years)	45.9/44.8	42.3/41.8	0.15	46.6/44.4	42.8/42.5	0.14
BMI (kg·m^−2^)	26.1/27.2	28.2/26.3	0.25	26.2/27.7	28/26.5	0.25
ME/CFS duration (years)	11.3/13.7	NA	NA	12.4/10.8	NA	NA
Bell disability scale	40.9/32.9	97.3/94.3	2 × 10^−16^ *	39.3/33.3	96.8/94.7	2 × 10^−16^ *

The means for each demographic parameter are shown for ME/CFS patients and controls. For the Bell disability scale, a higher number corresponds to better health. The *p*-values are from a Wilcoxon rank-sum test for the total cohort. The significance of *p*-values remains similar when segregated by sex. * indicates a significant difference between the ME/CFS and the control groups (*p*-value < 0.05). NA: not applicable.

## Data Availability

Almost all data is contained within the article and Supplementary Materials as well as at https://mapmecfs.org. Additional information about negative REAP assays can be obtained from Aaron Ring upon request.

## Supplementary Materials

The following supporting information can be downloaded at: https://www.mdpi.com/article/10.3390/ijms26062799/s1.
