# Supplementary material for: An In-Depth Exploration of the Autoantibody Immune Profile in ME/CFS Using Novel Antigen Profiling Techniques
Source: Int J Mol Sci. 2025 Mar 20;26(6):2799. doi: 10.3390/ijms26062799 (PMC11943395; doi:10.3390/ijms26062799)
Supplement: Supplementary file 1 [file ijms-26-02799-s001.zip › Supplemental Figures.pdf]

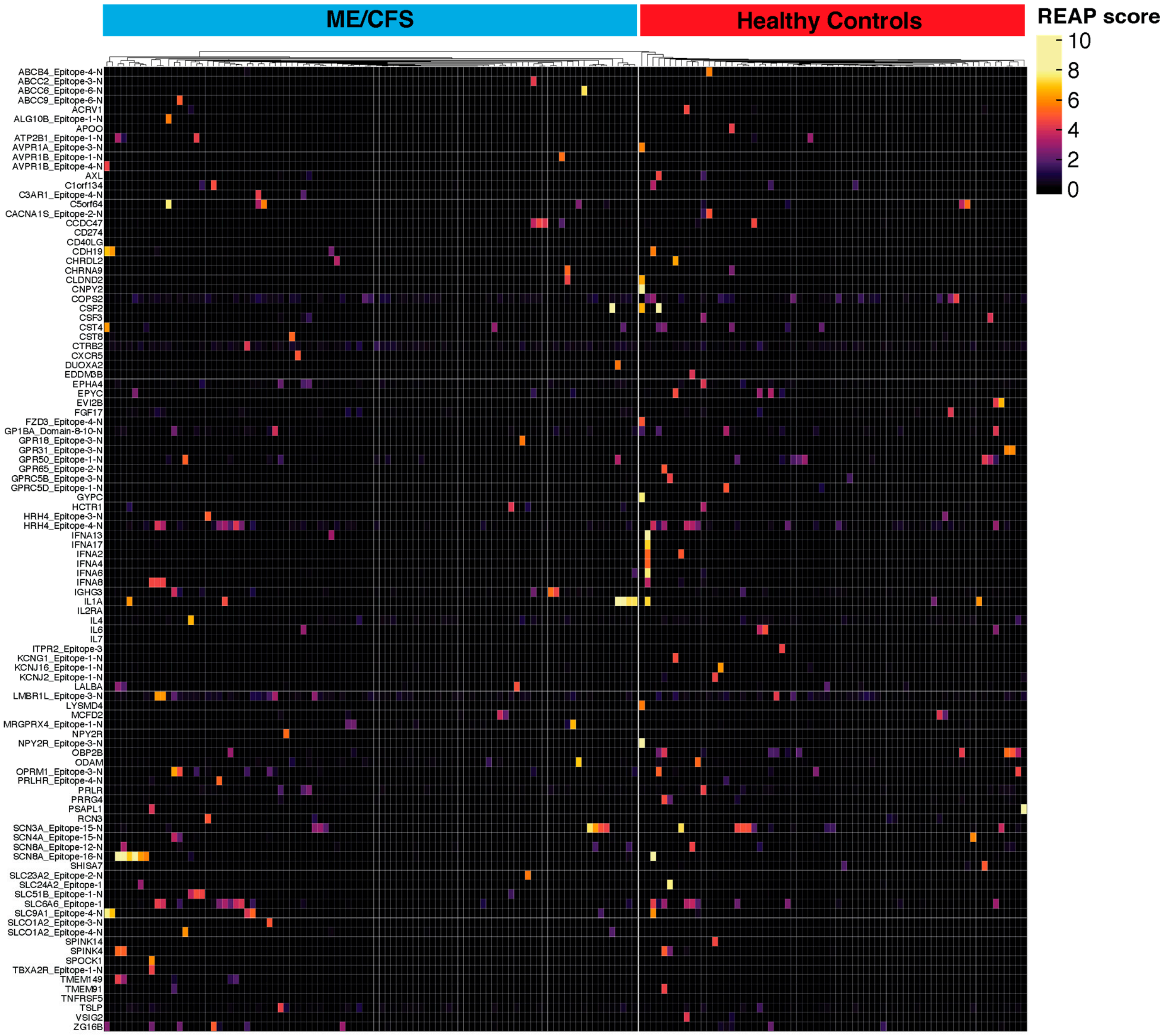

Figure S1. REAP reactivities of AAb across the ME/CFS and Healthy Controls cohorts – Column clustering was performed within groups by *k*-means clustering, $n = 69$ (HC), $n = 95$ (ME/CFS), with each column corresponding to one participant and each row representing one protein. Only reactivities with a REAP score ≥4 in at least one participant are shown.

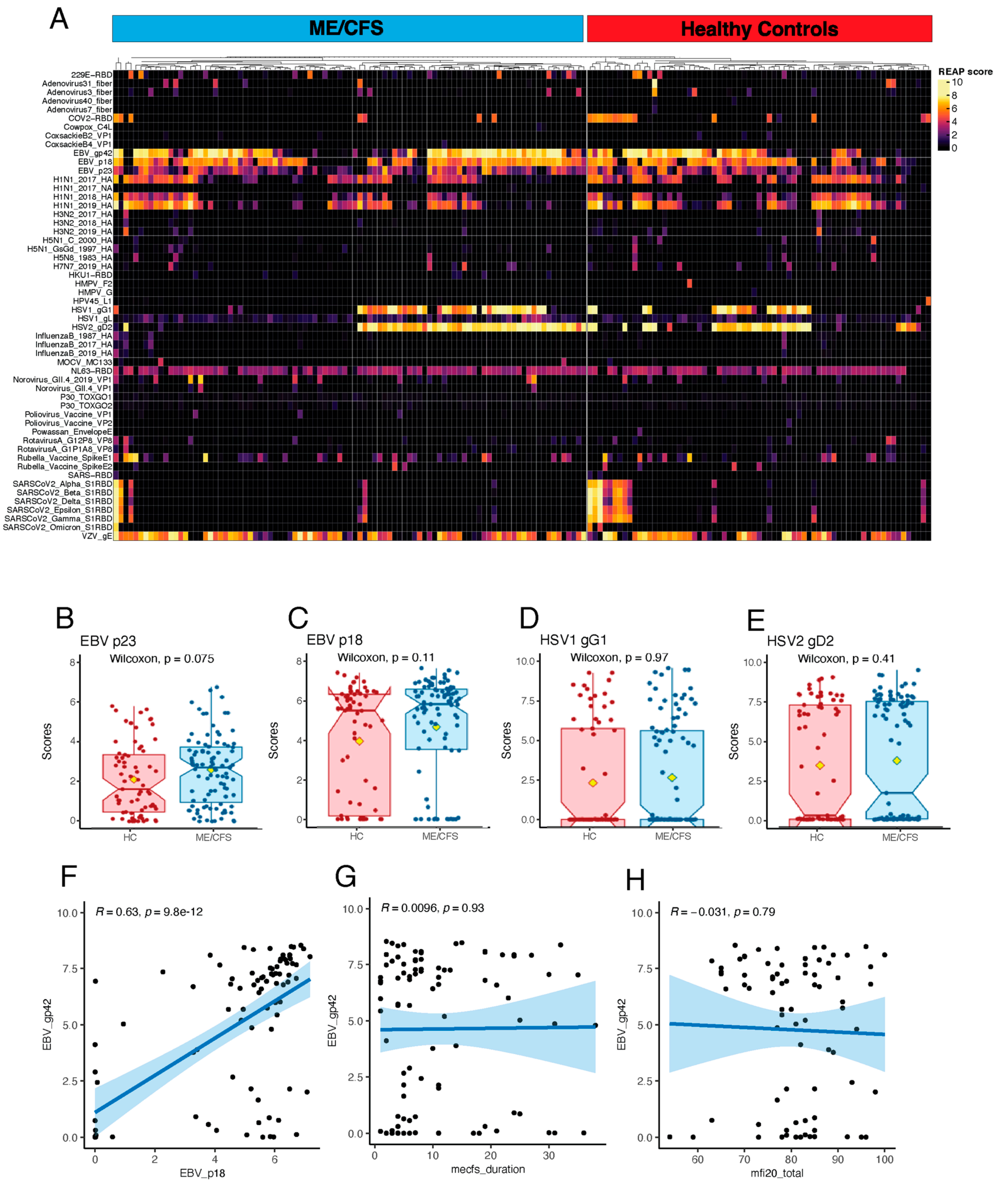

Figure S2. Viral antibodies – (A) REAP viral reactivities across the ME/CFS and HC cohort. Column clustering was performed within groups by *k*-means clustering, $n = 69$ (HC), $n = 95$ (ME/CFS), with each column corresponding to one participant and each row representing one protein. Only reactivities with a REAP score ≥0.5 in at least one participant are shown. **(B), (C), (D), (E)** REAP scores of healthy controls versus ME/CFS patients for EBV p23; EBV p18; HSV1 gG1; and HSV2 gD2 respectively. Statistical significance assessed by unpaired Wilcoxon rank-sum test. Each dot represents one individual. HC = healthy controls and ME = ME/CFS. The y-axis values are the values provided by the REAP panel. The yellow diamonds represent the mean. **(F), (G), (H)** Relationship between the REAP score of EBV gp42

and the REAP score and EBV p18; the REAP score of EBV gp42 and ME/CFS disease duration; and the REAP score of EBV gp42 and MFI-20. Correlation was assessed using Spearman's correlation. The blue line shows the linear regression, and the shading shows the 95% CIs. Each dot represents one individual.
